# Effects of dexmedetomidine on sleep quality of patients after surgery without mechanical ventilation in ICU

**DOI:** 10.1097/MD.0000000000007081

**Published:** 2017-06-08

**Authors:** Weina Lu, Qinghui Fu, Xiaoqian Luo, Shuiqiao Fu, Kai Hu

**Affiliations:** The Department of SICU, The Second Affiliated Hospital of Zhejiang University School of Medicine, Hangzhou, Zhejiang Province, China.

**Keywords:** BIS, dexmedetomidine, sedation, sleep depth

## Abstract

Sleep quality of patients in intensive care unit (ICU) has been recently recognized as an important aspect of the intensive care. Dexmedetomidine is one of the most recently introduced for sedation in the ICU. This study was designed to evaluate the effect of dexmedetomidine on sleep quality of patients without mechanical ventilation in ICU.

The patients who were included in this study were divided into two groups. In the sedation group, dexmedetomidine was given by a continuous infusion targeting a sedation level –1 to –2 on the score of RASS (Richmond Agitation-Sedation Scale). In the no sedation group, the patients slept by themselves. No other sedatives were given. Bispectral Index (BIS) was performed on these hemodynamically stable critically ill patients for 12 consecutive hours. Sleep time and sleep depth were recorded.

Twenty patients were studied. Compared to no sedation group, sleep efficiency and sleep time of patients in the sedation group was significantly higher during the night. Moreover, there was no significantly difference between the changes of blood pressure, heart rate, and respiratory rate.

Dexmedetomidine is a clinically effective and safe sedative for the highly selected critically ill patients without endotracheal tube and mechanical ventilation in the ICU to increases total sleep time and improve sleep efficiency.

## 1 Introduction

Sleep quality of patients in ICU has been recently recognized as an important aspect of the intensive care.^[1]^ Abnormalities of sleep are extremely common in critically ill patients, about half of total sleep time occurs during the daytime, and circadian rhythm is markedly diminished or lost.^[2]^ Therefore, patients are considered to have less-qualitative sleep in the ICU at night because of a stress environment in ICU. Moreover, the poor sleep quality can bring about prolonged ICU, hospital stay, delirium, long-term cognitive impairment, and increased morbidity. To maintain minimize stress, relieve pain and anxiety, sedation is essential for the treatment of patients in the intensive care unit. However, conventional sedatives are not suitable for patients without mechanical ventilation in the ICU because of the effect of respiration inhibition such as propofol and midazolam.^[3]^ Hence, α-2-adrenergic agonist without respiratory depression may be the choice for the patients without mechanical ventilation in the ICU.

Dexmedetomidine is one of the most recently introduced for sedation in the ICU.^[4]^ It is a new potent and highly selectivity α-2-adrenergic agonist, with the action of dose-dependent sedation, anti-anxiety, and assist-analgesia.^[5]^ It has been shown that dexmedetomidine more closely resemble natural sleep compared with other GABA (γ-aminobutyric acid) agonists in ICU sedation.^[6]^ Meanwhile, dexmedetomidine has little effect on influence on hemodynamics and provides a hemodynamic stability.^[7]^ In contrast to other sedatives, dexmedetomidine which can excite α2 receptors has analgesic effects without respiratory depression.^[8]^ Therefore, dexmedetomidine is safe and suitable for the patients without mechanical ventilation in the ICU.

Polysomnography is the gold standard for the analysis of sleep architecture but is not always available in routine practice, as it is time consuming and cumbersome for patients.^[9]^ The bispectral index (BIS) can be used as a practical and simplified tool to evaluate natural sleep depth. However, BIS only can be used to identifying stage N3 with satisfactory sensitivity and specificity but is limited by its inability to distinguish REM sleep from wake.^[10]^ Therefore, in our study, we only estimate sleep time, sleep depth, and awaking time by BIS.

The aim of this study was to investigate the function of dexmedetomidine about facilitating sleep quality and to evaluate the safety of dexmedetomidine for critically ill patients without mechanical ventilation in the ICU. In this study, we compared sleep time, sleep efficacy, cardiovascular responses (mean arterial pressure [MAP], heart rate), respiratory rate, and safety profile between dexmedetomidine sedation group and no sedation group.

## 2 Methods

### 2.1 Inclusion criteria and exclusion criteria for selective of patients

The study, performed between October 2015 and January 2016, was approved by the ethics Committee, the Second Hospital of Zhejiang University. All these patients were postabdominal surgery patients who did not need mechanical ventilation but still need stay in ICU for at least 3 days after study entry were enrolled. Inclusion criteria were (1) age >18 years and <65 years, (2) without mechanical ventilation and clinical need for therapy in SICU for at least 3 days after study entry were enrolled, (3) the patients were hemodynamically stable without sedative drugs before. Exclusion criteria were (1) patients with psychiatric disorders, (2) hypoxic-ischemic encephalopathy, (3) neurological disease and neurological injury that may effect on sleep quality, (4) severe hemodynamic instability, (5) severe abnormalities of cardiac conduction system (second or third-degree atrioventricular block) and severe ventricular dysfunction, (6) severe liver failure (bilirubin >100 μmol/L), (7) Glasgow Coma Scale less than 11, (8) acute physiology score portion of the Acute physiology and Chronic Health evaluation II (APACHe II) greater than 15.

### 2.2 Study protocol

Every patient was studied for a total of 12 hours, from 20:00 (the first day) to 8:00 (the second day). These patients were divided into 2 groups as the sedation group and no sedation group. In the sedation group, at 20:00 on the first night, dexmedetomidine was given by a loading dose from 0.2 to 0.7 μg /kg/h to maintain a sedation level –1 to –2 on RASS by micropump. In the no sedation group, no sedation medicine was used into these patients. As an institutional protocol, these patients in these 2 groups were receiving sufentanil to relieve pain stimulation in order to decrease the influence of pain to sleep. The score of NRS (numerical rating scale) which can assess ache grade were kept from 1 to 3. Noise and nursing operation were minimized during the nights of the study as far as possible. Moreover, during the night of study, light of ward was decreased to a minimum level that did not disturb with patients’ sleep. If the patient in the no sedation group suffered delirium and sleep disorder that there was need for sedation, the patient was withdrawn from this study. The bispectral index (BIS) was performed on each patient. Slept depth and sleep time of these 2 groups were continuous recorded by BIS (bispectral index) machine. The monitor machine of Covidien BIS VISTA was used in this study to record BIS value. The BIS index indicates the level of sedation and sleep depth, digitizing the EEG signal by the range of number from 0 to 100. Values from 85 to 100 represent wakefulness and values from 65 to 85 represent sedation or sleep status. The score of RASS was evaluated by nursing operation every 2 hours. Total sleep time, awakenings time of sleep, heart rate, blood pressure, oxyhemoglobin saturation, and respiratory rate were recorded during the night at 2-hour intervals. Sleep efficiency was calculated as the ratio between the effective sleep time and the total sleep time and expressed as percentage.

### 2.3 Statistical analysis

All data were recorded and noted on observation charts and were analyzed at the end of the study. Quantitative data of this study did not conform to normal distribution; therefore, data were analyzed using nonparametric tests. Variables were compared using the Wilcoxon signed-rank test. Continuous variables were expressed as medians (25th to 75th interquartile range). Differences were considered significant when *P* < .05. Analysis was carried out using the SPSS 18.0 software.

## 3 Result

Twenty-four patients were enrolled at first, but 4 patients did not complete the study and were excluded from the analysis. Two patients presented delirium. Two patients suffered operative complications and had a second surgery. None of the patients had detectable levels of propofol. In this study, 11 patients received sedation with dexmedetomidine and 9 patients slept by themselves. These 20 patients were all postsurgical. Demographic data, such as gender, APACHE II scores, weight, age, child-pugh, and operation method were recorded. Baseline characteristics of these 20 patients are shown in Table 1.

**Table 1:**
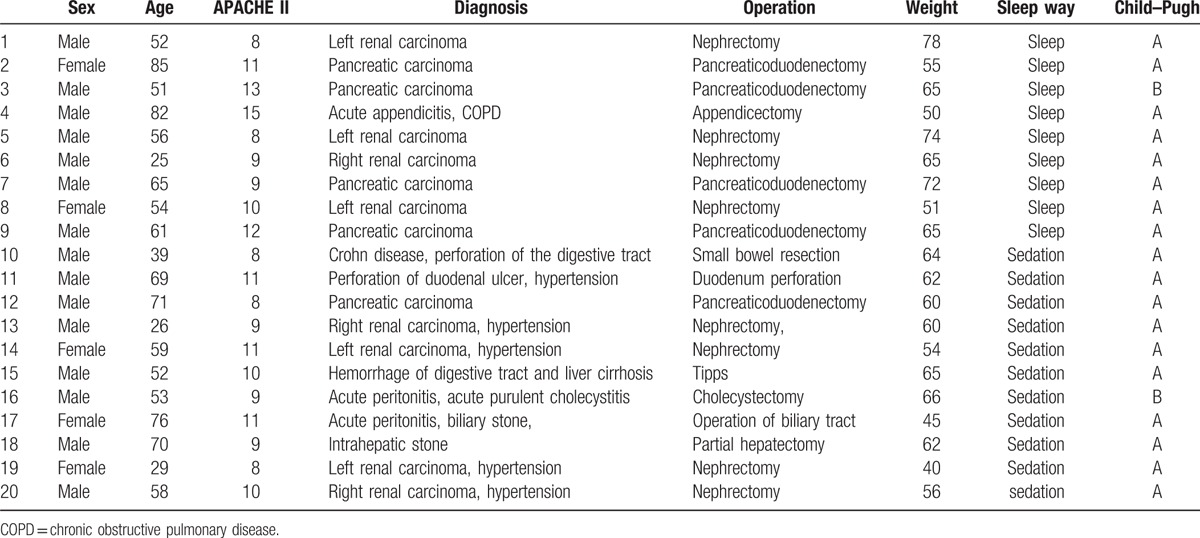
Demographic profile of patients.

**Figure 1:**
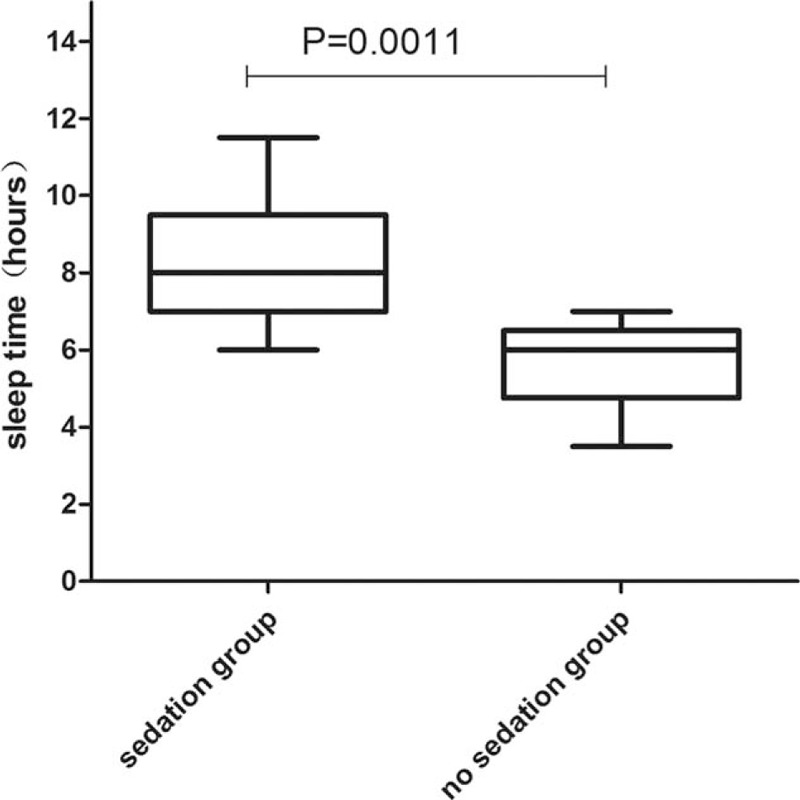
Box-whisker plot of sleep time.

**Figure 2:**
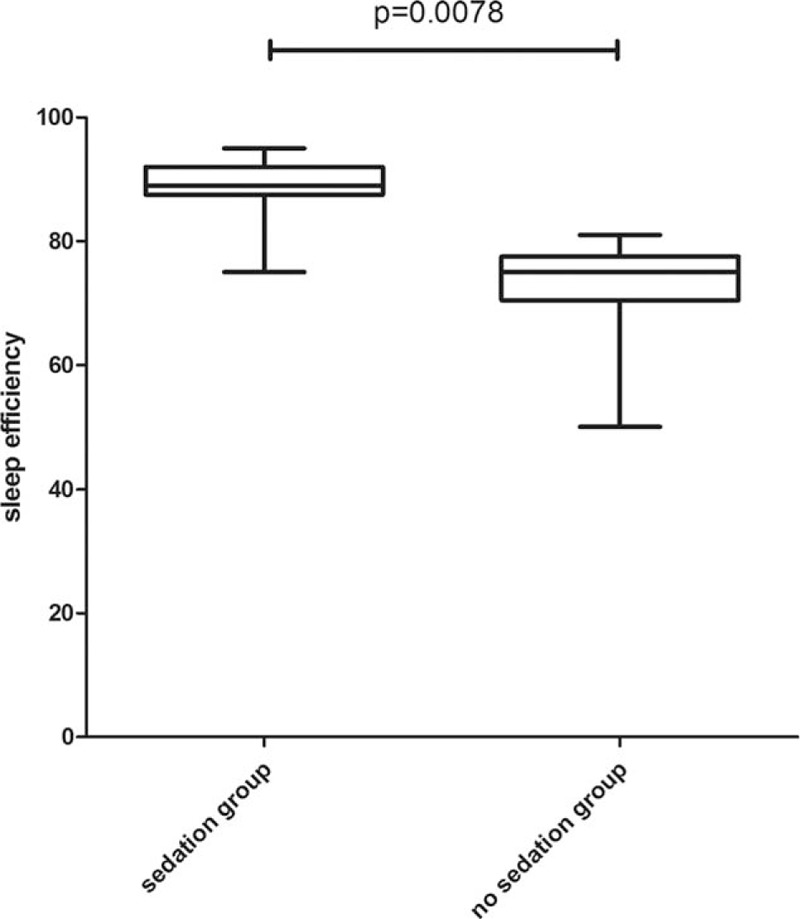
Box-whisker plot of sleep efficiency.

**Figure 3:**
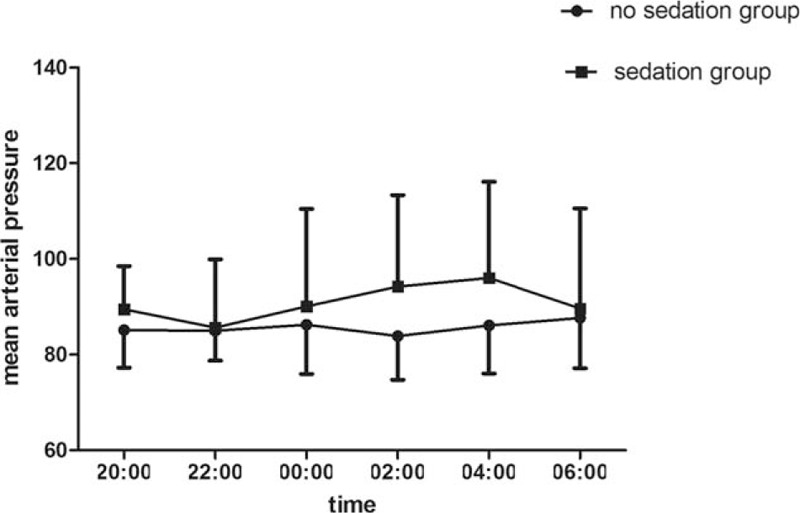
The trend of MAP (mean ± standard error of the mean) between dexmedetomidine infusion and no sedation group. MAP = mean arterial pressure.

**Figure 4:**
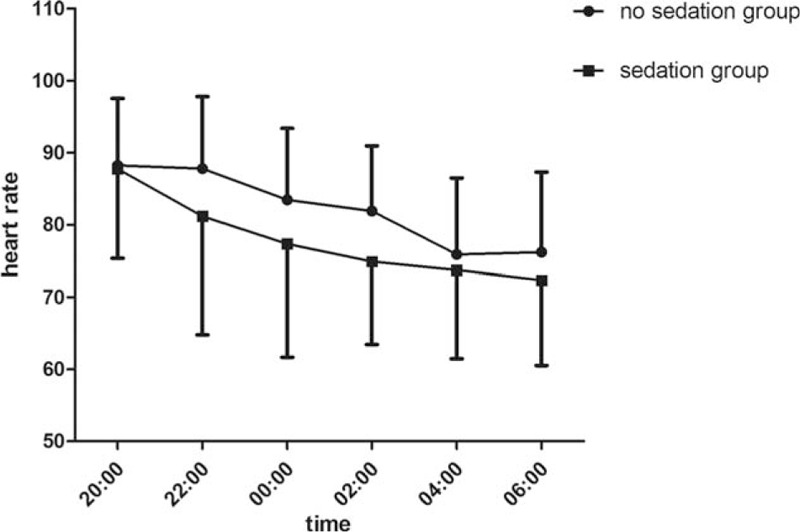
The trend of heart rate (mean ± standard error of the mean) between dexmedetomidine infusion and no sedation group.

**Figure 5:**
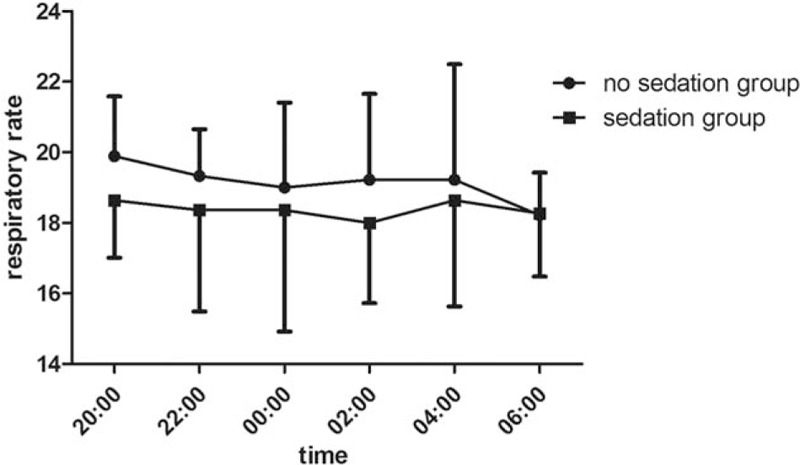
The trend of respiratory rate (mean ± standard error of the mean) between dexmedetomidine infusion and no sedation group.

There is no statistical significance between 2 groups in age, height, and weight. Throughout the entire night from 20:00 to 8:00, oxyhemoglobin saturation, blood pressure, and respiratory rate remained stable and without significant differences between 2 groups which were showed in Table 2. There is statistical significance between 2 groups in sleep efficiency and total sleep time. Compared to group without dexmedetomidine infusion, sleep efficiency and sleep time were significantly higher in the group with dexmedetomidine sedation (Figs. 1 and 2). The baseline hemodynamic parameters were near in both groups. No significant change in MAP from the baseline occurred in the sedation group. MAP had presented minimal changes in both groups. Thereafter, mean values of MAP in both groups remained in a stable range throughout study period from 22:00 to 8:00 (Fig. 3). Patients received dexmedetomidine had significantly lower heart rates than patients slept by themselves. Patients who received dexmedetomidine also had significantly lower heart rates from baseline in 20:00 (Fig. 4). In these 2 groups, no significantly difference about respiratory rates. Patients received dexmedetomidine did not have respiratory depression compared to no sedation group (Fig. 5). The values of respiratory rate in the dexmedetomidine group and no sedation group were statistically insignificant in our study. Moreover, the oxygen saturation in the dexmedetomidine group was found not to reduce lower than 97%.

**Table 2:**
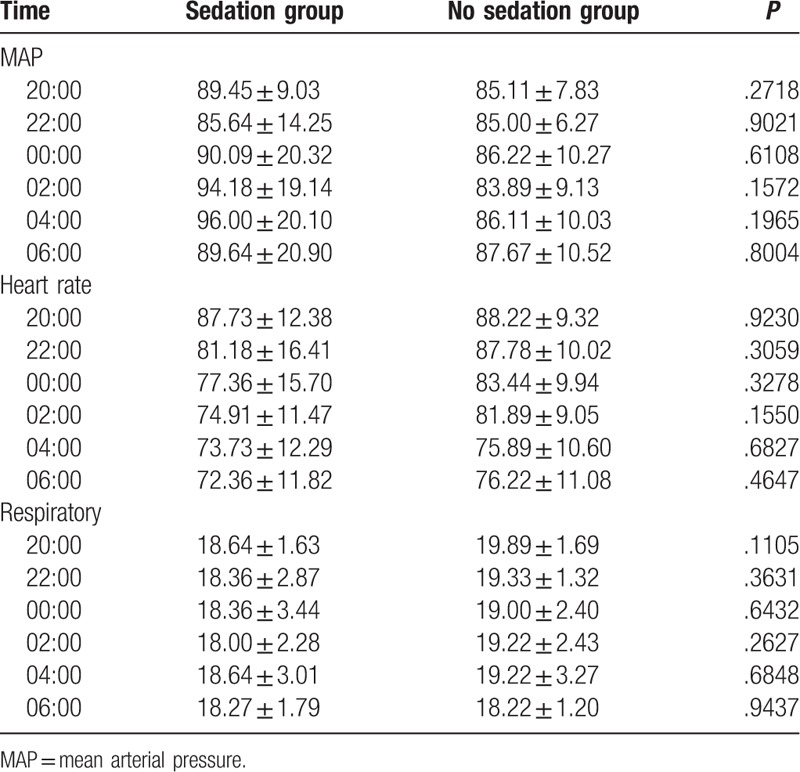
Comparison of MAP, heart rate, and respiratory rate between 2 groups.

## 4 Discussion

The chief results of this study showed that in critically ill patients without mechanical ventilation, the use of dexmedetomidine during the night to achieve light sedation (RASS –1 to –2) significantly increased sleep efficiency. Moreover, the patients in the sedation group had stable hemodynamically and no respiratory depression compared with patients in no sedation group slept by themselves. Many previous studies have confirmed that dexmedetomidine is a safe and effective medicine for ICU sedation of patients without respiratory depression and reduces the incidence of delirium in critically ill patients.^[11]^

Compared with GABA agonists, dexmedetomidine produces its pharmacologic effect through exciting a-2 receptor, it has minimal effects on cognitive impairment.^[12]^ In addition, dexmedetomidine produces a state called “cooperative sedation.” This dexmedetomidine-induced sedation is similar to endogenous sleep pathways.^[13]^ Meanwhile, dexmedetomidine does not have respiratory depressive effects so that it is safe for the patients without mechanical ventilation in the ICU. Some studies had been shown that compared to GABA agonists, dexmedetomidine more closely resembles natural non-ReM sleep, which facilitated the patient–caregiver interaction.^[14,15]^

The ICU ward is in a stress environment. The level of noise in ICU is comparable to that in a factory or a busy office and is louder than noise in a bedroom.^[16]^ Acute illness, pain, light, and patient discomfort are all factors that contribute to sleep abnormalities in critically patients.^[17]^ Sleep quality in ICU is poor because of above factors. Therefore, morbidity and mortality of critically ill patients can be influenced by sleep fragmentation and sleep deprivation.^[18,19]^ Our result of this study is similar to those reported^[20]^ that critically ill patients in the ICU presented poor quality sleep and short sleep time. As a result, sedative which can facilitate sleep quality is necessary for the patients in ICU. From our study, dexmedetomidine infusion by micropump to achieve the recommend sedation depth in critically ill patients without mechanical ventilation increased sleep efficiency and the total sleep time. A study reported by Christina Alexopoulou,^[21]^ who observed that critically ill patients exhibit disorganized and poor quality sleep as evidenced by the lack of sequential progression through sleep stages and low percentages of SWS (slow wave sleep) and REM sleep (rapid eye movement sleep). The stage of NREM and REM is recorded by polysomnography in the study by Christina Alexopoulou. Polysomnography is the gold standard for the analysis of sleep architecture but is not always available in routine practice, as it is time consuming and cumbersome for patients. BIS can identify stage N3 with satisfactory sensitivity and specificity but is limited by its inability to distinguish REM sleep from wake.^[10]^ Therefore, the BIS value was only used to record sleep time and sleep depth in our study.

Hypotension and bradycardia are both the side-effects of a-2 agonists.^[22]^ However, there are some previous studies showed that hemodynamic stability was preserved in most patients who received dexmedetomidine. A study reported by Uma Srivastava,^[7]^ who observed that dexmedetomidine had a better cardiovascular safety profile than clonidine, though dexmedetomidine and clonidine are both a-2 agonists. In our study, heart rate reductions happened in the sedation group, but this parameter stayed within clinically acceptable ranges. Meanwhile, there was not obviously change of blood pressure between the sedation group and no sedation group. This is similar to a study reported by Martin et al,^[5]^ who observed that continuous dexmedetomidine infusions did not increase the risk of cardiovascular complications in those patients with presurgical histories of hypotension, hypertension, bradycardia, or tachycardia. Unlike other sedatives, dexmedetomidine does not have side effect of respiratory depression and it does not affect the respiratory drive. Therefore, dexmedetomidine is appropriate for the patients without endotracheal tube and mechanical ventilation.

There are several limitations in this research that need further discussion. First of all, because of the strict selection criteria, the rate of recruitment was very low, so this was a small, single-center study. Second, because of short study time and ICU stay, whether or not delirium rate can alleviate was unknown. Third, because there was not a guideline about which value range in BIS can accurately indicate the sleep stage (REM and NREM), we cannot know whether dexmedetomidine can affect SWS and ReM sleep, so we only can record sleep time, sleep depth, and calculate sleep efficacy to evaluate sleep quality.

In conclusion, our results show that dexmedetomidine is a clinically effective and safe sedative for the highly selected critically ill patients without endotracheal tube and mechanical ventilation in the ICU to increases total sleep time and improve sleep efficiency. Moreover, we observed incidence of hypotension and bradycardia stayed within clinically acceptable ranges and respiratory depression did not happen. Therefore, dexmedetomidine is appropriate for the critically ill patients without endotracheal tube and mechanical ventilation in the ICU.
